# Comparison between four published definitions of hyposmia in Parkinson's disease

**DOI:** 10.1002/brb3.2258

**Published:** 2021-06-30

**Authors:** Sofia Kanavou, Vanessa Pitz, Michael A. Lawton, Naveed Malek, Katherine A. Grosset, Huw R. Morris, Yoav Ben‐Shlomo, Donald G. Grosset

**Affiliations:** ^1^ Population Health Sciences Bristol Medical School University of Bristol Bristol UK; ^2^ Institute of Neuroscience and Psychology University of Glasgow Glasgow UK; ^3^ Department of Neurology Queen's Hospital Romford Essex UK; ^4^ Institute of Neurological Sciences Queen Elizabeth University Hospital Glasgow UK; ^5^ Department of Clinical and Movement neuroscience UCL Queen Square Institute of Neurology London UK

**Keywords:** cut‐off, diagnosis, hyposmia, olfactory impairment, Parkinson's disease

## Abstract

**Objectives:**

Hyposmia is a common feature of Parkinson's disease (PD), yet there is no standard method to define it. A comparison of four published methods was performed to explore and highlight differences.

**Materials and methods:**

Olfactory testing was performed in 2097 cases of early PD in two prospective studies. Olfaction was assessed using various cut‐offs, usually corrected by age and/or gender. Control data were simulated based on the age and gender structure of the PD cases and published normal ranges. Association with age, gender, and disease duration was explored by method and study cohort. Prevalence of hyposmia was compared with the age and gender‐matched simulated controls. Between method agreement was measured using Cohen's kappa and Gwet's AC1.

**Results:**

Hyposmia was present in between 69.1% and 97.9% of cases in *Tracking Parkinson's* cases, and between 62.2% and 90.8% of cases in the *Parkinson's Progression Marker Initiative*, depending on the method. Between‐method agreement varied (kappa 0.09–0.80, AC1 0.55–0.86). The absolute difference between PD cases and simulated controls was similar for men and women across methods. Age and male gender were positively associated with hyposmia (*p* < .001, all methods). Odds of having hyposmia increased with advancing age (OR:1.06, 95% CI:1.03, 1.10, *p* < .001). Longer disease duration had a negative impact on overall olfactory performance.

**Conclusions:**

Different definitions of hyposmia give different results using the same dataset. A standardized definition of hyposmia in PD is required, adjusting for age and gender, to account for the background decline in olfactory performance with ageing, especially in men.

## Graphical abstract

There are multiple definitions of hyposmia in Parkinson's disease (PD), and subtle differences between them can lead to different and often non‐comparable results. To illustrate the need for a standardised definition of hyposmia in PD, that would adjust for age and gender and account for the background decline in olfactory performance with ageing, this paper compares four published definitions of hyposmia, demonstrating the effect of each definition on the research outcomes when applied to the same cohort.

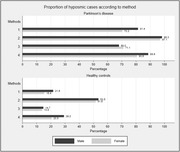

## 1 INTRODUCTION

Parkinson's disease (PD) is a progressive neurodegenerative movement disorder with core clinical motor features of bradykinesia in combination with rest tremor, rigidity, or both (Hughes et al., 1992). Nonmotor symptoms in PD are increasingly recognized. Olfactory impairment (hyposmia), rapid eye movement (REM) sleep disorder, constipation, and depression are common prodromal features of PD (Berg et al., 2015); the absence of hyposmia is a red flag (but not an exclusion criterion) in the clinical diagnostic criteria for PD (Postuma et al., 2015). Olfactory dysfunction occurs in a range of neurodegenerative diseases, including PD at motor presentation (Silveira‐Moriyama et al., 2009), prodromal PD (Barber et al., 2017; Campabadal et al., 2019; Lo et al., 2021; Noyce et al., 2014; Siderowf et al., 2012), progressive supranuclear palsy (Silveira‐Moriyama et al., 2010), and Alzheimer's disease (Jung et al., 2019), but olfaction is generally normal in multiple system atrophy (Xia & Postuma, 2020) and *Parkin*‐related PD (Malek et al., 2016). However, hyposmia also occurs with advancing age in healthy people and is more common in males than females (Doty et al., 1984; Stern et al., 1994), making its diagnostic use in PD challenging. Previously, the performance on olfactory testing in PD was considered to be constant and independent of disease duration (Doty et al., 1988), but recent evidence suggests that it declines with disease duration (Berendse et al., 2011). Over the years, there has been extensive research aiming to establish a standard method to define hyposmia, including the use of PD probability curves (Picillo et al., 2014; Silveira‐Moriyama et al., 2009), analysis of area under the receiver operator characteristic curve (Baba et al., 2012; Bohnen et al., 2008; Rodriguez‐Violante et al., 2014), and categorizing readings below a certain centile level (Ponsen et al., 2004), typically the 15th percentile (Pont‐Sunyer et al., 2015; Siderowf et al., 2012; Sierra et al., 2013).

The two most commonly applied tests of olfaction are the University of Pennsylvania Smell Identification Test (UPSIT) which has 40 odors identified from scratch‐and‐sniff panels and Sniffin’ Sticks (SS) which has 16 odors identified from uncapped pens. Both tests have a forced choice option from four odors and have published normative data centiles stratified by age and gender, and results can be combined using established equivalence methods (Lawton et al., 2016).

There is a wide range of results reported for the prevalence of hyposmia in PD. Some of this heterogeneity is likely to be due to lack of standardization. Several methods have been used to define abnormal olfaction, often involving adjusting for age, gender, or both. It is likely that variation in the analytical methods has contributed to the wide range of reported hyposmia in PD of between 74.3% and 100% (Szewczyk‐Krolikowski et al., 2014; White et al., 2016).

Our aim was to compare four established methods that have been previously applied in PD research (Doty, 2008; Noyce et al., 2014; Siderowf et al., 2012; Silveira‐Moriyama et al., 2009) to define the rates of hyposmia in early PD, and compare this to normative data. We sought to empirically highlight the effect of using those definitions, thereby aiding interpretation of past studies, as well as informing decision making in the interpretation of olfaction testing in future clinical research.

## 2 MATERIALS AND METHODS

### 2.1 Data sources

Olfaction test results were analyzed from two longitudinal cohorts of recent onset Parkinson's disease, Tracking Parkinson's, a UK multicenter prospective study of cases diagnosed within the preceding 3.5 years, and the *Parkinson's Progression Markers Initiative* (*PPMI*), a United States led multicountry study of newly diagnosed cases (Marek et al., 2011). All patients had a clinical diagnosis of PD, fulfilling UK Brain Bank criteria (Malek et al., 2015), or supported by abnormal presynaptic dopaminergic imaging (Hughes et al., 1992; Marek et al., 2011).

Olfaction was tested in *Tracking Parkinson's* at 6 months after recruitment, using either the British 40‐item version of UPSIT, or the 16‐item SS, and in *PPMI* at study entry applying the 40‐item US version of UPSIT. UPSIT is a “scratch‐and‐sniff” test for 40 odors with a forced choice from four options per odor, and gives a maximum score of 40 (Doty, 2008). The Sniffin’ test consists of 16 odors from a “smell pen”, again with a forced choice from four items per odor, and a maximum score of 16 (Hummel et al., 1997). We converted UPSIT to SS scores using an algorithm developed from item response theory, as previously reported (Lawton et al., 2016).

### 2.2 Definitions of hyposmia

We used four definitions of hyposmia as follows: (a) Method 1 (age‐corrected) defines patients aged 60 years or older as hyposmic when UPSIT score is below 24, while patients aged under 60 years are hyposmic when the score is below 29 (Silveira‐Moriyama et al., 2009). (b) Method 2 (gender‐corrected, using absolute UPSIT values) creates an ordinal variable that also incorporates severity of hyposmia; this defines males scoring below 19 as anosmic, between 19 and 25 as severely hyposmic, between 26 and 29 as moderately hyposmic, between 30 and 33 as mildly hyposmic, and above 34 as normosmic. For females, anosmia and severe hyposmia have the same cut‐offs, while moderate hyposmia is a score of 26–30, mild hyposmia is 31–34, and scores above 35 are normosmic. This method can also be used to create a binary cut‐off for hyposmic versus normosmic (Doty, 2008). (c) Method 3 (age and gender corrected) defines hyposmia as UPSIT scores at or below the 15th centile based on normative data obtained from healthy individuals (Siderowf et al., 2012). We applied an extension of this definition, using “smoothed” cut‐points as previously demonstrated (Lawton et al., 2016). (d) Method 4 (percentile method) uses a score below the 15th centile of the population studied to define hyposmia (Noyce et al., 2014) as implemented in the PREDICT‐PD study, where eligible healthy participants aged 60–80 years, identified in part through the Parkinson's UK membership list had an olfactory assessment. Participants scoring at or below 27 in UPSIT were classified as hyposmic (“at‐risk”).

### 2.3 Other variables

The two study cohorts were compared in terms of motor severity, measured by the Movement Disorder Society Unified Parkinson's Disease Rating Scale (MDS‐UPDRS part 3); level of cognitive impairment by the Montreal Cognitive Assessment (MoCA); and disease stage by Hoehn and Yahr.

### 2.4 Statistical analysis

Differences in clinical and demographic characteristics between study cohorts were tested by an independent samples *t*‐test for continuous variables; Mann‐Whitney test for MoCA score, UPDRS part 3 score, and Hoehn and Yahr stage; chi‐square test for binary variables and Kruskal‐Wallis for the graded hyposmic status derived from Method 2. Between method agreement was measured using Cohen's kappa (κ) which is widely used to compare observations and methods, and “corrects” for chance agreement. Since kappa can be affected by prevalence of the index condition (e.g., hyposmia), resulting in paradoxically low or high values (Feinstein & Cicchetti, 1990), we also measured agreement using Gwet's AC1, which also has the advantage of not requiring independence between tests (Gwet, 2008). A general rule of thumb proposed by Koch and Landis (Landis & Koch, 1977) suggests that negative values indicate no agreement, whereas between 0−0.20 indicates slight, 0.21−0.40 fair, 0.41−0.60 moderate, 0.61−0.80 substantial, and 0.81−1 an almost perfect agreement.

Six different outcome measures were investigated. A binary outcome was used (hyposmic versus normosmic) for each of the four definitions stated above (Methods 1–4). We also examined olfaction as an ordinal outcome (anosmic, severe, moderate, mild hyposmia, and normosmic) as defined in Method 2 above, and finally using olfaction as a continuous variable with a range from 0 to 16 on the raw or converted SS scale.

For each of the four binary outcomes, we used logistic regression and adjusted each model appropriately: for disease duration and gender in Method 1 as age was already accounted for; for disease duration and age in Method 2 as gender was accounted for; for disease duration in Method 3 as both age and gender were accounted for; and for age, gender, and disease duration in Method 4. For the ordinal outcome, we used an ordered logistic regression model and the Brant test to check the assumption that the odds are proportional across the different levels of the outcome (known as the parallel regression assumption). For the continuous outcome, simple linear regression was used to assess the relation between olfactory performance and gender, age, and disease duration at the date of the test.

We also created a simulated “normative” dataset by applying the data reported for healthy controls by age and gender (Doty, 2008) to the age and gender structure of the *Tracking Parkinson's* cohort, in order to derive what would be expected (the “counterfactual”) to be observed if this cohort did not have PD. The statistical software R version 3.3.3 and StataMP version 15.1 were used.

## 3 RESULTS

**FIGURE 1:**
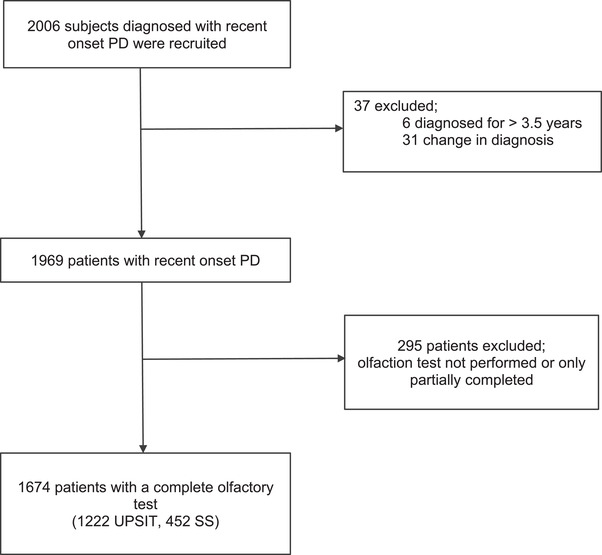
Patient disposition for olfaction testing in the *Tracking Parkinson's* cohort. Abbreviations: PD, Parkinson's disease; UPSIT, University of Pennsylvania Smell Identification Test; SS, Sniffin’ Sticks. Patients recruited to the study were excluded from analysis for the reasons shown. One patient took both tests but only UPSIT results were used. In the majority of the 295 cases who did not complete olfaction testing, a complete loss of sense of smell was reported

In *Tracking Parkinson's* out of 2006 patients initially recruited to the study 1674 cases completed olfaction testing (Figure 1), 73% using UPSIT and 27% using the SS test. In *PPMI* there were 423 cases, all of whom performed the UPSIT test. Cases in *Tracking Parkinson's* were significantly older, by around 6 years at the time of olfaction testing and had lower cognitive and olfaction scores compared to *PPMI* cases (Table 1). The gender distribution and motor severity were very similar between the cohorts, as well as the overall disease staging. The proportion of hyposmic participants varied from 62.2% to 97.9%. The prevalence of hyposmia was highest when using Method 2, followed by Method 4. Methods 1 and 3 showed similar results in *PPMI* but produced higher estimates in *Tracking Parkinson's*. The effect of age and gender adjustment for the 15th centile (Method 3) had the biggest effect on reducing the intercohort differences (69.1% versus 63.6%).

**TABLE 1:** Demographic and clinical characteristics of 2097 patients with early Parkinson's disease who performed an olfactory test

Characteristic		*Tracking Parkinson's*(*N* = 1674)	*PPMI*(*N* = 423)	*p*‐value
Male gender		1085 (64.8%)	277 (65.5%)	.796
Age at diagnosis (years)		65.9 (9.2)	61.1 (9.7)	<.001
Age at time of test (years)		67.8 (9.2)	61.6 (9.7)	<.001
Disease duration at time of test (years)		1.9 (0.9)	0.6 (0.5)	<.001
MDS UPDRS 3		20.0 (14.0 ‐ 29.0)	20.0 (14.0 – 26.0)	.247
Montreal cognitive assessment		26.0 (23.0 ‐ 28.0)	28.0 (26.0 – 29.0)	<.001
Hoehn and Yahr stage^†^	0 to 1.5	805 (48.5%)	185 (43.7%)	.553
	2 or 2.5	758 (45.6%)	236 (55.7%)	
	3+	98 (5.9%)	2 (0.5%)	
UPSIT total score^‡^		19.7 (6.8)	22.3 (8.2)	<.001
SS total score^§^		7.4 (3.0)	–	–
Prevalence of hyposmia by method				
Method 1: Age‐corrected cut‐offs		1297 (77.5%)	263 (62.2%)	<.001
Method 2: Absolute values	Any grade	1639 (97.9%)	384 (90.8%)	<.001
	Mild	101 (6.2%)^¶^	47 (12.2%)	<.001
	Moderate	207 (12.6%)^¶^	69 (18.0%)	
	Severe	480 (29.3%)^¶^	121 (31.5%)	
	Anosmic	851 (51.9%)^¶^	147 (38.3%)	
Method 3: 15th centile (age and gender‐corrected)		1157 (69.1%)	269 (63.6%)	.180
Method 4: 15th centile (uncorrected)		1436 (85.8%)	298 (70.4%)	<0.001

Abbreviations: MDS UPDRS, Movement Disorder Society Unified Parkinson's Disease Rating Scale.; PPMI, Parkinson's Progression Markers Initiative; SS, Sniffin’ Sticks test; UPSIT, University of Pennsylvania Smell Identification Test.

Data are given as mean (SD); median (interquartile range) for continuous variables; and percentage for categorical variables.

^†^ 13 cases had missing values.

^‡^ Out of 1222 patients in Tracking Parkinson's, and 423 patients in PPMI, who took the test.

^§^ Out of 453 patients who took the test.

^¶^ Percentages of hyposmic cases.

**FIGURE 2:**
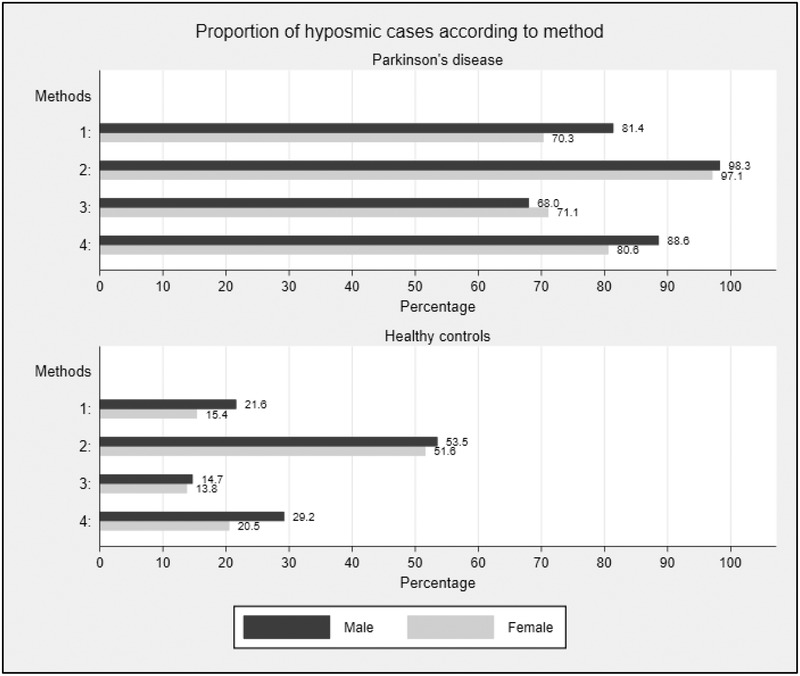
Prevalence of hyposmia in patients with early Parkinson's disease and simulated healthy individuals of matching age and gender. Percentages of hyposmic cases shown at the end of the bars

Regardless of method, the prevalence of hyposmia was higher in the PD cases than in the normative simulated control population (Figure 2 and Table 2). Unsurprisingly, Method 2 resulted in the highest percentage of PD patients being classified as hyposmic and the same was true for simulated controls, followed by Method 4, 1, and then 3. This was seen in both men and women. Both Methods 2 and 3 reduced any gender differences for the PD and simulated control data. The differences in the proportion of hyposmia between cases and controls was largest for women (60.1%) using the 15th centile uncorrected method (Table 2) and was similar for men (59.4% and 59.8%) using the 15th centile uncorrected method and the age‐corrected cut‐offs. Method 2 showed the smallest differences between PD and control subjects.

**TABLE 2:** Comparisons of hyposmia rates by gender in patients with early Parkinson's disease and simulated healthy individuals of matching age and gender

	Males			Females		
Method	Parkinson's	Non‐PD	Difference (%)	Parkinson's	Non‐PD	Difference (%)
1: Age‐corrected cut‐offs	81.4 (79.0, 83.6)	21.6 (19.2, 23.9)	59.8	70.3 (66.5, 73.8)	15.4 (12.7, 18.5)	54.9
2: Absolute values						
Any grade	98.3 (97.4, 99.0)	53.6 (51.0, 56.2)	44.7	97.1 (95.4, 98.2)	51.6 (47.9, 55.3)	45.5
Mild	5.2 (4.0, 6.7)	18.6 (16.3, 21.2)	−13.4	7.6 (5.7, 10.1)	23.6 (20.0, 27.0)	‐16
Moderate	10.4 (8.7, 12.4)	10.6 (8.8, 12.4)	−0.2	16.0 (13.2, 19.1)	10.5 (8.0, 13.1)	5.5
Severe	26.3 (23.8, 29.1)	12.2 (10.4, 14.2)	14.1	32.9 (29.2, 36.8)	8.0 (5.8, 10.2)	24.9
Anosmic	56.4 (53.4, 59.3)	12.2 (10.3, 14.2)	44.2	40.6 (36.7, 44.6)	9.4 (7.1, 11.7)	31.2
3: 15th centile, corrected for age and gender	68.0 (65.2, 70.7)	14.7 (12.7, 17.0)	53.3	71.1 (67.4, 74.7)	13.8 (11.0, 16.8)	57.3
4: 15th centile, uncorrected	88.6 (86.5, 90.3)	29.2 (26.9, 31.7)	59.4	80.6 (77.2, 83.6)	20.5 (17.5, 23.6)	60.1

Data are percentage (95% empirical CI).

Abbreviation: PD = Parkinson's disease.

Levels of agreement among the four methods varied (Table 3). Cohen's kappa ranged between 0.09 and 0.66 in *Tracking Parkinson's* and 0.29–0.80 in *PPMI;* whereas Gwet's AC1 estimates were closer between the two cohorts (*Tracking Parkinson's*: 0.60–0.86; *PPMI*: 0.55–0.83). In some comparisons, kappa was substantially lower than Gwet's AC1 regardless of the cohort, for example in Method 1 versus Method 2 gave a kappa of 0.14 versus an AC1 of 0.74 in *Tracking Parkinson's*, whereas in *PPMI* the difference was smaller (Kappa = 0.29; AC1 = 0.55). Kappa and Gwet's AC1 were as expected closer in the comparison of methods where the categorization used similar cut‐offs, for example Method 1 versus Method 3, in both cohorts.

**TABLE 3:** Agreement among four methods of classifying PD patients as hyposmic using Kappa and Gwet's AC1 measures of agreement

	2: Absolute values^†^		3: 15th centile (age‐ and gender‐ corrected)		4: 15th centile (uncorrected)	
	Cohen'sKappa	Gwet'sAC1	Cohen'sKappa	Gwet'sAC1	Cohen'sKappa	Gwet'sAC1
			Tracking Parkinson's cohort			
1: Age‐corrected cut‐off points	0.14 (0.10, 0.18)	0.74 (0.71, 0.77)	0.63 (0.59, 0.68)	0.76 (0.73, 0.79)	0.66 (0.61, 0.70)	0.85 (0.83, 0.87)
2: Absolute values^a^			0.09 (0.06, 0.12)	0.60 (0.56, 0.64)	0.23 (0.16, 0.29)	0.86 (0.84, 0.88)
3: 15th centile (age‐ and gender‐ corrected)					0.45 (0.40, 0.49)	0.69 (0.66, 0.72)
			PPMI cohort			
1: Age‐corrected cut‐off points	0.29 (0.21,0.36)	0.55 (0.47, 0.63)	0.80 (0.74, 0.86)	0.83 (0.77, 0.88)	0.74 (0.68, 0.81)	0.79 (0.73, 0.85)
2: Absolute values			0.30 (0.22, 0.38)	0.58 (0.50, 0.66)	0.39 (0.30, 0.48)	0.70 (0.64, 0.77)
3: 15th centile (age‐ and gender‐ corrected)					0.58 (0.50, 0.66)	0.67 (0.59, 0.74)

Abbreviation: PD, Parkinson's disease; PPMI, Parkinson's Progression and Markers Initiative.

^†^ In Method 2 different grades of hyposmia are merged.

In *Tracking Parkinson's*, the continuous olfactory data had normal residuals with a slight negative skewness (left tail). We found that male gender (*p* < .001), increasing age (*p* < .001) and longer disease duration (*p* = .01) had a negative impact on patients' overall olfactory performance when using the continuous score in our linear regression model (Table 4). Disease duration was not significant in any of the logistic regression models (Method 1: *p* = .27, Method 2: *p* = .69, Method 3: *p* = .10, Method 4: *p* = .37). However, both older age (Methods 2 OR 1.06, 95% CI 1.03, 1.10, *p* < .001 and 4 OR 1.05 95% CI 1.03, 1.06, *p* < .001) and male gender (Method 1 OR 1.85, 95% CI 1.47, 2.34, *p* < .001 and Method 4 OR 1.82, 95% CI 1.38, 2.42, *p* < .001) predicted hyposmia. Ordered logistic regression also showed that disease duration (*p* = .01) and age (*p* < .001) predicted hyposmia and the proportionality assumption was not violated (*p* = .68). Very similar results were seen with the *PPMI* cohort (Table 4) with age and gender predicting olfaction and there being no real association with disease duration while noting that the sample size was lower than in *Tracking Parkinson's*.

**TABLE 4:** Statistical analysis results for each study cohort

	Disease duration		Age		Gender (male)	
Analysis models:	Model estimate (95% CI)	*p*‐value	Model estimate (95% CI)	*p*‐value	Model estimate (95% CI)	*p*‐value
	Tracking Parkinson's cohort					
Linear regression	−0.20^†^ (−0.35, −0.06)	.01	−0.09^†^ (−0.10, −0.07)	<.001	−0.99^†^ (−1.28, −0.70)	<.001
Logistic regression						
1: Age‐corrected cut‐points	1.07^‡^ (0.95, 1.21)	.27	–	–	1.85^‡^ (1.47, 2.34)	<.001
2: Absolute values (gender‐corrected)	0.93^‡^ (0.65, 1.33)	.69	1.06^‡^ (1.03, 1.10)	<.001	–	–
3: 15th centile, corrected for age & gender	1.10^‡^ (0.98, 1.23)	.10	–	–	–	–
4: 15th centile, uncorrected	1.07^‡^ (0.92, 1.25)	.37	1.05^‡^ (1.03, 1.06)	<.001	1.82^‡^ (1.38, 2.42)	<.001
Ordered logistic regression	1.14^‡^ (1.03, 1.25)	.01	1.06^‡^ (1.05, 1.07)	<.001	–	–
	PPMI cohort					
Linear regression	0.42^†^ (−0.95, 1.79)	.55	−0.26^†^ (−0.34, −0.18)	<.001	−2.61^†^ (−4.17, −1.05)	.001
Logistic regression						
1: Age‐corrected cut‐points	0.85^‡^ (0.59, 1.21)	.36	–	–	1.59^‡^ (1.06, 2.40)	.03
2: Absolute values (gender‐corrected)	0.79^‡^ (0.45, 1.40)	.43	1.05^‡^ (1.02, 1.09)	.002	–	–
3: 15th centile, corrected for age & gender	0.85^‡^ (0.59, 1.21)	.37	–	–	–	–
4: 15th centile, uncorrected	1.08^‡^ (0.72, 1.63)	.71	1.07^‡^ (1.04, 1.09)	<.001	1.81^‡^ (1.16, 2.84)	.01
Ordered logistic regression	0.95^‡^ (0.69, 1.31)	.77	1.06^‡^ (1.04, 1.08)	<.001	–	–

Abbreviation: PPMI, Parkinson's Progression Markers Initiative.

^†^ Beta coefficient given as model estimate.

^‡^ Odds ratios given as model estimate.

## 4 DISCUSSION

The classification of cases as hyposmic, or the diagnosis of hyposmia, based on clinic‐based olfaction testing, shows substantial variability according to the method applied. Given the association of patients’ age and gender with olfactory performance, and despite the choice of analysis outcome (continuous test scores or hyposmic/normosmic binary status), correction for age and gender is strongly advised when assessing the level of olfactory impairment in PD.

Using the *Tracking Parkinson's* dataset to analyze all four methods, our results showed that between 68% and 98% of PD patients are defined as hyposmic. Moreover, hyposmia was present in a substantial proportion of healthy individuals, between 14% and 54% depending on the method applied. As expected from such wide ranges, agreement between test methods was limited according to both the kappa statistic, and the alternative Gwet's AC1 measure. It is therefore not surprising that different studies add further variability according to the type of olfaction testing method, the age and gender ratios of patients studied, as well as the disease duration in the population studied, in findings regarding the application of olfactory testing in Parkinson's disease (Gaig et al., 2014; White et al., 2016).

Both kappa and the robust Gwet's AC1 coefficient showed substantial agreement between classifying subjects with scores below the 15th centile as hyposmic (Method 4) or using an age‐corrected cut‐off in *Tracking Parkinson's* (kappa = 0.66 vs AC1 = 0.85) and *PPMI* (kappa = 0.74 vs AC1 = 0.79). A moderate to substantial agreement was evident between Method 4 and using an age‐ and gender‐corrected 15th centile (Method 3) to classify PD patients (Table 3).

Our study findings also confirmed that olfactory ability of PD patients is associated with age and gender. Sense of smell deteriorates with advancing age, and the odds of a patient who is a year older to be hyposmic increased by 5%–7% in both *Tracking Parkinson's* and *PPMI* according to Methods 2 and 4. Similarly, we found fairly consistent evidence showing greater hyposmia in men compared to women. Thus, when comparing two populations, whether they involve PD or non‐PD cases, unless they have been matched on age and gender, it is necessary to do some form of adjustment unless the definition of hyposmia has already taken this into account, as in Method 3. Otherwise, differences in the proportion of subjects who are classified as hyposmic may differ due to confounding introduced by these two variables.

We found some evidence of a modest association between hyposmia and longer disease duration. There was a significant association between disease duration and olfactory performance in two of the models in *Tracking Parkinson's* (the linear regression model and ordered logistic regression model) but not in any of the logistic regression models. This may reflect the loss of power when using a dichotomous rather than ordinal or continuous outcome (Altman & Royston, 2006). Similarly, none of the models in *PPMI* found an effect of disease duration but again power may be an issue, as *PPMI* had around 25% of the sample size compared to *Tracking Parkinson's* and both cohorts have limited variation in disease duration. Ideally, one would measure olfaction repeatedly as disease duration increases and model this in relation to longitudinal changes in the non‐PD population.

The strength of our analyses is that we have used two independent cohorts and a variety of methods of defining hyposmia based on the published literature to demonstrate how different definitions can modify results. One of the limitations is the combination of two different olfactory tests (SS, UPSIT) although there is evidence of the effectiveness of the conversion method we have used (Lawton et al., 2016). Neither study had a gold or reference standard test to compare the screening criteria with, but this is usually the case in large scale prospective studies of PD. It remains possible that a more detailed olfactory assessment than odor identification (which we employed) involving odor threshold testing and odor discrimination (Hummel et al., 1997), could prove more sensitive and/or specific in differentiating between PD and healthy controls, but these tests are more time‐consuming and seldom performed in large clinical studies.

In conclusion, standardized testing and the use of consistent statistical modelling for olfactory impairment can help in identifying hyposmia as a risk marker for PD, therefore refining at‐risk cohorts in clinical studies.

## ETHICS STANDARDS AND INFORMED CONSENT

All participating sites from both studies received approval from an ethical standards committee on human experimentation before study initiation and in accordance with the Declaration of Helsinki and the Good Clinical Practice (GCP) guidelines. Written informed consent was obtained from all patients included in the study.

### PEER REVIEW

The peer review history for this article is available at https://publons.com/publon/10.1002/brb3.2258.

## CONFLICT OF INTEREST

SK, VP, MAL, NM, KAG, and YBS have no conflict of interest

## FUNDING STATEMENT

HRM: Dr Morris is employed by UCL. In the last 24 months he reports paid consultancy from Biogen, UCB, Abbvie, Denali, Biohaven, Lundbeck; lecture fees/honoraria from Biogen, UCB, C4X Discovery, GE‐Healthcare, Wellcome Trust, Movement Disorders Society; Research Grants from Parkinson's UK, Cure Parkinson's Trust, PSP Association, CBD Solutions, Drake Foundation, Medical Research Council. Dr Morris is a co‐applicant on a patent application related to C9ORF72 ‐ Method for diagnosing a neurodegenerative disease (PCT/GB2012/052140).

DGG: Dr Grosset received honoraria from Bial Pharma, Merz Pharma, and consultancy fees from The GM Clinic, Glasgow.

PPMI – a public‐private partnership – is funded by the Michael J. Fox Foundation for Parkinson's Research and funding partners, including AbbVie, Allergan, AVid, Biogen, BioLegend, Bristol‐Myers Squibb, Celgene, Jenali, GE Healthcare, Genentech, GlaxoSmithKline, Lilly, Lundbeck, Merck, Meso Scale Discovery, Pfizer, Piramal, Prevail Therapeutics, Roche, Sanofi Genzyme, Servier, Takeda, Teva, UCB, Verily, Voyager Therapeutics.

## Data Availability

The data that support the findings of this study are available on request from the corresponding author. The data are not publicly available due to privacy or ethical restrictions.
